# Uncovering Competitive and Restorative Effects of Macro- and Micronutrients on Sodium Benzoate Biodegradation

**DOI:** 10.3389/fmicb.2021.634753

**Published:** 2021-03-17

**Authors:** Purvi Zaveri, Aishwarya Raghu Iyer, Rushika Patel, Nasreen Shakil Munshi

**Affiliations:** Institute of Science, Nirma University, Ahmedabad, India

**Keywords:** textile effluents, macronutrients, micronutrients, sodium benzoate biodegradation, *Pseudomonas citronellolis*, response surface methodology

## Abstract

A model aromatic compound, sodium benzoate, is generally used for simulating aromatic pollutants present in textile effluents. Bioremediation of sodium benzoate was studied using the most abundant bacteria, *Pseudomonas citronellolis*, isolated from the effluent treatment plants of South Gujarat, India. Multiple nutrients constituting the effluent in actual conditions are proposed to have interactive effects on biodegradation which needs to be analyzed strategically for successful field application of developed bioremediation process. Two explicitly different sets of fractional factorial designs were used to investigate the interactive influence of alternative carbon, nitrogen sources, and inorganic micronutrients on sodium benzoate degradation. The process was negatively influenced by the co-existence of other carbon sources and higher concentration of KH_2_PO_4_ whereas NH_4_Cl and MgSO_4_ exhibited positive effects. Optimized concentrations of NH_4_Cl, MgSO_4_, and KH_2_PO_4_ were found to be 0.35, 1.056, and 0.3 mg L^–1^ respectively by central composite designing. The negative effect of high amount of KH_2_PO_4_ could be ameliorated by increasing the amount of NH_4_Cl in the biodegradation milieu indicating the possibility of restoration of the degradation capability for sodium benzoate degradation in the presence of higher phosphate concentration.

## Introduction

India is the second largest textile product-exporting country worldwide, and textile sector plays a major role in the employment, economic growth, and financial empowerment to millions of Indians from smll handicraft units to large apparel industries. Gujarat is a major textile-manufacturing hub in the country. Textile industries utilize huge amount of fresh water for production of finished goods. Effluents generated from fabric printing, yarn printing, and dyeing can cause considerable amount of damage to the environment due to the presence of colors, salts, and a variety of recalcitrant compounds (Ayed et al., 2012). Years of research has indicated the importance and cost effectiveness of biological treatment over physicochemical method for textile wastewater treatment. However, bioremediation as a bioprocess when applied to field becomes one of the most dynamic and complex interplay of pollutants, nutrients, and microorganisms. Microbial community present in contaminated environment (e.g., soil, water, wastewater) generally has efficient microbial degraders like *Pseudomonas* species in adequate numbers (Rossello-mora et al., 1994; Junca and Pieper, 2004). Such highly versatile organisms are found to have an ability to metabolize complex/toxic hydrocarbons like phenol, toluene, and phenanthrene under laboratory conditions (Lakshmi et al., 2013). The question arises, with so diverse metabolic capability why such efficient microbial community fails to generate the laboratory-evident degradation rate under field conditions? The limitation lies in our understanding to generate conducive environment, interplay of nutrients, translation of bioremediation to field, and strategic optimization of bioprocess developed in laboratory. To understand the interaction and interference of various macro- and micronutrient on biodegradation, statistical optimization was employed using a simplest model hydrocarbon such as sodium benzoate (SB) which was used to mimic aromatics in textile wastewater (Yaseen and Scholz, 2019), by *P. citronellolis* (GenBank accession number: KM871063) (Zaveri et al., 2015). Under various environmental conditions, sodium benzoate has been reported to travel through route of wastewaters and end up in sludge (Wibbertmann et al., 2005). Though it is easily biodegradable under controlled laboratory conditions (Wibbertmann et al., 2005), reports also suggest that the presence of preferable carbon and/or nitrogen sources (glucose, acetate, amino acids, etc.) result in either increase or decrease in hydrocarbon biodegradation efficiency (Chun et al., 2004; Jonsson et al., 2007; Das and Chandran, 2011). Hence, in the presence of easily assimilable carbon sources, hydrocarbon compounds will be least preferred and may not be degraded. Similarly, even if the organisms have the capacity to degrade such hydrocarbon compounds, but in absence of suitable nitrogen sources, the compounds remain undegraded.

It is known that deficiency of required inorganic nutrients may lead to lower biodegradation rates which may be a result of nutrient starvation leading to degradation of cellular proteins (John and Goldberg, 1980; Walworth et al., 2007). However, there are reports which also suggest that supplementing the depleted inorganic nutrients may not be the stimulus each time as these metabolic processes are dependent on interplay of compounds and/or organisms (Swindoll et al., 1988; Steffensen and Alexander, 1995). Thus, optimization of bioprocess turns out to be the key factor for processes like enzyme production as well as biodegradation to achieve high biodegradation efficiency (Dibblef and Bartha, 1979; Leahy and Colwell, 1990; Yan et al., 2014; Priyadharshini and Bakthavatsalam, 2016; Hashem et al., 2018).

With increased access to sophisticated statistical software and computing, research field witnesses large application of Design of Experiment (DOE) mathematical tool for bioprocess engineering. Statistical designing of experiments has been shown to be a very important tool in optimizing various media components for industrial scale processes such as production of enzymes (Abdel-Fattah et al., 2005; Yuan et al., 2008; Demir et al., 2015), biodegradation of various toxic compounds (Östberg et al., 2007; de Guillén-Jiménez et al., 2012; Ibn Abubaker et al., 2012; Lakshmi et al., 2013; Su and Lin, 2013), biodegradation in marine environment (Zahed et al., 2010), bacterial biomass production (Gutierrez-Rojas et al., 2011; Zhang et al., 2014; Chen et al., 2015), etc. As these tools have the capability to display interactive influential effect of variables, they have replaced conventional one-variable-at-a-time approach (OVAT) for multivariant experiments. Plackett-Burman design (PBD), a fractional factorial design, determines critical/significant variables affecting the processes in a limited number of experiments (Burman and Plackett, 1946). This design helps to illustrate and identify the effect of each parameter on the bioprocess and their interaction with each other (El-Hamid et al., 2018). It has also been found to be helpful in attaining increased gene expression and enzyme production (Abdel-Fattah et al., 2007; Halder et al., 2013; Meng et al., 2015). The screened variables/components significantly affecting the biodegradation process can then be further optimized using response surface methodology (RSM) (Darvishmotevalli et al., 2019). For a generation of response plots, various designs like central composite design (CCD), Box-Behnken design, etc. are widely applied.

In the present study, field condition was simulated for degradation of single-ring hydrocarbon with the help of statistical tools for characterization of main effect of variables, i.e., nutrients, on SB biodegradation process by *Pseudomonas citronellolis*. Initially, in our studies on sodium benzoate degradation by *P. citronellolis*, few variables were optimized using OVAT approach including pH, substrate concentration, temperature, aeration-agitation, and nitrogen source. Furthermore, 9 variables were identified which were supposed to influence SB degradation. To reduce the number of experiments required for optimizing the rest of the 9 parameters for SB degradation, experiments were designed using fractional factorial design of Plackett-Burman. Out of 11 variables selected in the PB design, two variables were having known effects, i.e., substrate concentration and pH obtained from independent studies using OVAT approach. In two explicitly different sets of PBD for studying the interaction of carbon and nitrogen sources in degradation experiments, the importance of the presence of micronutrients was realized. In addition, a detailed analysis of desirable concentration and influence of variables on each other is described using 3D response plots. Such detailed approach can be used as platform for interactive analysis of other combinations of nutrients, as the robustness of the design helps the researcher to conclude the most influential variable easily. The results obtained indicate the obstacles with degradation of target hydrocarbon in the presence of easily degradable substrates which might be available in surrounding environment. The primary aim of using PBD was to evaluate the influence of additional “C” source selected arbitrarily as supplementary nutrients for growth of bacterial cells used for bioremediation or various “N” sources for screening their effects on SB degradation and interaction with other media constituents. The study deals with the nutrient interplay involved in sodium benzoate degradation and does not focus on the mass balance and mineralization thus stoichiometry of the benzoate degradation process and pathways are not in the scope of the present study. Moreover, no simulation software/AI was used to generate any biological data, and the results are outcome of SB degradation experiments conducted under laboratory conditions.

## Materials and Methods

### Materials

All chemicals used in this study were of the highest purity grade purchased from Sigma-Aldrich, Merck-India, or Himedia-India.

### Microorganism and Culture Activation

The bacterial strain used in this study, *Pseudomonas citronellolis* was isolated from common industrial effluents of South Gujarat, India and was identified as the most abundant bacteria in effluent stream in our previous work (Zaveri et al., 2015). Activated culture grown on nutrient broth (OD_560_ □0.8) was harvested at 7,500 rpm at 27 ± 2°C temperature (Eppendorf Centrifuge Model No. 5430 R, Germany). To ensure removal of organic matter, activated culture was washed twice with sterile normal saline before inoculation (5%, *v*/*v*) in autoclaved media prepared for PBD and RSM experiments as described in the section “Statistical Design of Experiments.”

### Statistical Design of Experiments

The objective of applying statistical techniques was to reduce the number of optimization experiments to deduce the most optimal conditions for SB degradation using limited number of experiments with statistical validity.

#### Plackett-Burman Design: Fractional Factorial Experiment

The effects of carbon and nitrogen sources and their interactions with micronutrients on SB degradation were assessed independently in two experimental set ups designed using PBD. Design Expert (ver. 10) was used to prepare run combinations for all experiments described here. Design A was formulated for evaluation of the presence of more than one *carbon sources* and design B was a representation of condition where the presence of more than one *nitrogen sources* was evaluated. Essential micronutrients (KH_2_PO_4_, Na_2_HPO_4_, MgSO_4_, and CaCl_2_) were kept as common variables in both the designs although the “−1” concentrations were kept intentionally different (with difference of one level) so that they do not become the limiting factor in runs with longer incubation period. Concentration of all other variables in both the designs were kept at “−1” (low) and “+1” (high) levels. The concentrations and the details of the variables used are presented in Table 1. The two levels of all factors (concentration of “+1” and “−1”) were selected in such a way as to match either the concentration of ingredients of minimal salt medium (MSM) (Atlas, 2005) or which helps us to further select the concentrations to be used in five-level experimental design of response surface methodology using CCD.

**TABLE 1:** Details of variables used for Plackett-Burman designing for optimization of sodium benzoate degradation.

Nutrient type	Sr. No.	Design A				Design B	
		Factor (code)	Concentration levels (mg)		Factor (code)	Concentration levels (mg)	
			−1	+1		−1	+1
Micronutrients	1	KH_2_PO_4_ (H)	300	400	KH_2_PO_4_ (A)	200	400
	2	Na_2_HPO_4_ (G)	1,200	1,800	Na_2_HPO_4_ (B)	600	1,800
	3	NaCl (J)	800	1,000	NaCl (C)	600	1,000
	4	MgSO_4_ (B)	1.23	1.56	MgSO_4_ (D)	0.738	1.722
	5	CaCl_2_ (C)	0.0735	0.1029	CaCl_2_ (E)	0.041	0.102
Macronutrients	6	Succinate (D)	500	700	Glycine (G)	75	125
	7	Acetate (E)	500	700	Proline (H)	75	125
	8	Glucose (F)	500	700	Cysteine (J)	75	125
	9	NH_4_Cl (A)	500	700	Ammonium tartrate (L)	100	300
	10	pH (L)	6.5	7	Paranitrophenol (K)	1.5	2.5
	11	Sodium benzoate (K)	5 mM	7 mM	Sodium benzoate (F)	5 mM	7 mM

The code designations of variables were kept random to avoid any bias in design. A total of 11 variables were considered for investigation, where 9 were independent variables and some were used as dummy variables (unassigned variables) viz. SB concentration and pH in design A and only SB concentration in design B. They were screened in 12 combinations/runs according to the design. In design A, factors 1–5 were ingredients of modified MSM [Na_2_HPO_4_,600 mg L^–1^; KH_2_PO_4_, 300 mg L^–1^; NH_4_Cl, 100 mg L^–1^; NaCl, 50 mg L^–1^; MgSO_4_, 24.6 mg L^–1^; CaCl_2_, 1.4 mg L^–1^; and SB, 72 mg L^–1^ (5 mM)] selected for hydrocarbon degradation assay. Factors 6, 7, and 8 were selected as additional substrates/“C” source to investigate their role as supplementary/co-existing substances to investigate whether they may support growth of more number of cells and thus may potentially enhance SB degradation. Also, whether they are interacting with other constituents of medium could become clear in such experiments. Similarly, in the case of design B, factors 1–5 were the constituents of modified minimal salt basal medium and factors 6–9 were the nitrogen sources selected to be screened (Table 1). All the runs were performed in triplicates with sufficient negative controls. According to described concentrations, 100 ml media were prepared and inoculated with active, washed culture (5%, *v*/*v*) of *P. citronellolis* and incubated at 30 ± 2°C under shaking conditions (80 rpm) (Remi CIS-24 plus, India). Initial pH of the medium was adjusted using 0.1 N HCl or NaOH. Responses were calculated in terms of percent SB degradation from average values of replicates in each run (Rajendran et al., 2007; Patil and Jena, 2015; El-Hamid et al., 2018).

The main effect plot was prepared, and using Pareto chart, the influential factors were identified as those exhibiting values above “t-critical’ and Bonferroni’s limit.

#### Central Composite Design: Response Surface Methodology

As from the results of designs “A” and “B,” micronutrients viz. NH_4_Cl, MgSO_4_, and KH_2_PO_4_ were found to have a significant effect on SB degradation bioprocess; they were then further considered for model development using CCD through RSM. A three-factor, five-level CCD with 20 runs was employed (Safa et al., 2017). The variables NH_4_Cl, MgSO_4_, and KH_2_PO_4_ were denoted as factors 1, 2, and 3, respectively, and each of them were assessed at five different levels, combining factorial points (−1, + 1), axial points (−*α*, + *α*), and central point (0). The concentration used for matrix designing using CCD is projected in Table 2 (Priyadharshini and Bakthavatsalam, 2016). Twenty runs were required according to Design Expert (ver. 10.0), and run details are presented as Supplementary Material. According to levels of all three factors, with respective concentrations of these ingredients, media were prepared in 100 ml volumes, sterilized, and inoculated with 5% (*v*/*v*) *P. citronellolis* culture. Responses were calculated as SB degradation percentages obtained from experimental sets performed in triplicates. The rest of all the components of modified MSM were kept to their original concentrations including 5 mM of sodium benzoate as sole source of carbon.

**TABLE 2:** Selected levels and concentrations of variables used in central composite design for applying response surface methodology.

Factors	Levels				
	−*α*	−1	0	+ 1	+*α*
KH_2_PO_4_	0.131	0.200	0.300	0.4	0.468179
NH_4_Cl	0.163	0.300	0.500	0.7	0.83636
MgSO_4_	0.402	0.738	1.230	1.722	2.05744

The predicted values as obtained through modeling were compared with actual values obtained through the experiment. Three-dimensional response plots and their respective contour plots for all three micronutrients were prepared using Design Expert (Ver. 10.0) and analyzed to obtain the desirable concentration for better SB remediation.

### Monitoring of SB Degradation

All flasks were incubated at 30 ± 2°C in an incubator shaker at 80 rpm. At regular intervals, 1 ml sample was aseptically withdrawn to determine growth of organism and degradation of sodium benzoate. Changes in pH values were observed using universal pH indicator (Himedia, India product code-I013); however, it was found to be negligible. Remaining sodium benzoate concentration was determined spectrophotometrically at 230 nm (Agilent Technologies Carry 60 UV-Vis, United States) (Zaveri et al., 2015). Abiotic loss of SB during incubation was less than 2% and thus, all the degradation values were normalized accordingly. Percent SB degradation was calculated using the following formula:

(1) $$\begin{array}{c} \mathrm{SB}\mathrm{degradation}\left(\%\right)=[(\mathrm{Initial}\mathrm{SB}\mathrm{concentration}-\mathrm{Final}\mathrm{SB}\\ \mathrm{concentration})/\mathrm{Initial}\mathrm{SB}\mathrm{concentration}]\times 100 \end{array}$$

All the degradation experiments were performed in triplicates, and the results presented are average of three data sets.

## Results

Initial studies for sodium benzoate degradation were conducted to optimize variables over a possible range for individual parameter. This data contributed to the basic understanding of degradation pattern and characteristic of organism under variously stressed conditions. The “One Variable at a Time” approach was used for basic parameters like temperature (15, 20, 25, 30, and 40°C), pH (4, 5, 6, 7, 8, 9, and 10), agitation (0, 80, and 150 rpm), nitrogen source (NH_4_Cl and KNO_3_), and substrate concentrations (ranging from 1 to 100 mM). The results obtained indicated that organism was able to optimally utilize 1–50 mM sodium benzoate using NH_4_Cl as source of nitrogen at 30°C and at 80 rpm. The organism could not withstand pH 4 and 10 and substrate concentrations above 50 mM. The organism was able to degrade substrate under both static and agitating conditions; however, agitation was supporting faster degradation as compared with static conditions. From the range of substrate concentrations, 5 and 7 mM were selected for factorial designing because of the least possible degradation time required and the lowest sensitive detection with spectrophotometric method.

Statistical methods were used only to design the experimental combinations of nutrients, based on which flask-level experiments were conducted. No data reported in the present manuscript is software derived. All the data for SB degradation were obtained after conducting wet lab experiments at flask level.

### Identification of Major Effect in Two Independent Plackett-Burman Experiments

The experimental response obtained in the form of SB degradation through both the designs according to the change in the medium composition is depicted in Table 3. In the modified MSM medium, *Pseudomonas citronellolis* could degrade SB by more than 95% within 24 h of incubation. With the change in the composition of the medium, degradation percentage ranged from 0 to 68% (incubation till 18 days) in design A and 40–87% degradation in design B (incubation till 6 days). The wide range of values obtained indicates prominent effect of multiple carbon and nitrogen sources in the medium and interaction of variables on degradation of sodium benzoate.

**TABLE 3:** Response of SB degradation by *Pseudomonas citronellolis* in two PB designs for medium constituents.

Run number	SB degradation (%)	SB degradation (%)
	Design A	Design B
1	69 ± 6	85 ± 2
2	65 ± 2	78 ± 2
3	56 ± 8	77 ± 2
4	59 ± 9	76 ± 1
5	57 ± 3	83.6
6	59 ± 2	78 ± 2
7	0.0	82 ± 1
8	39 ± 1	40 ± 1
9	63 ± 5	55 ± 4
10	0.0	85 ± 1
11	6 ± 6	86 ± 6
12	0	83 ± 1

**FIGURE 1:**
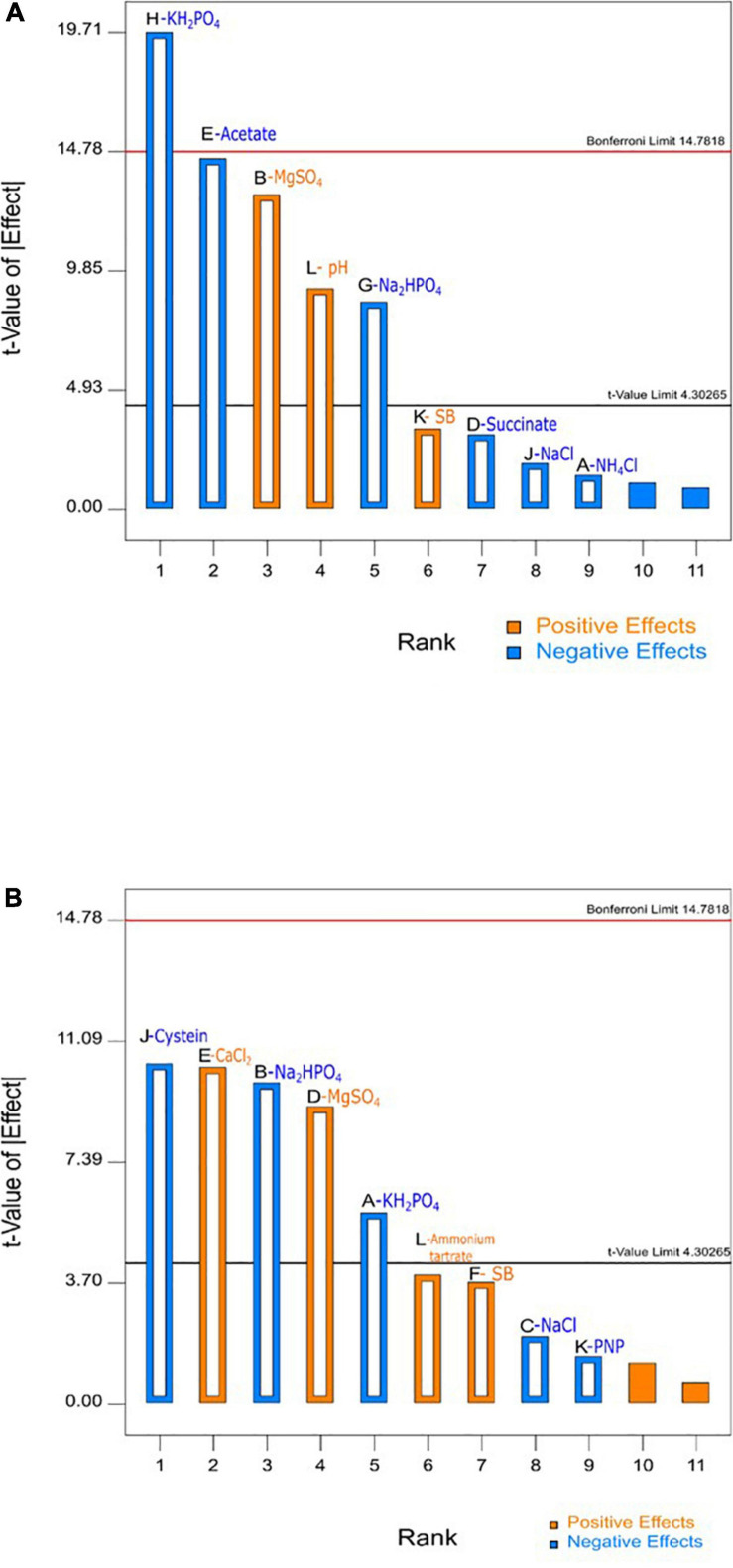
Pareto chart for obtaining main effects using fractional factorial **(A)** design A and **(B)** design B conducted for sodium benzoate degradation by *P. citronellolis.*

**FIGURE 2:**
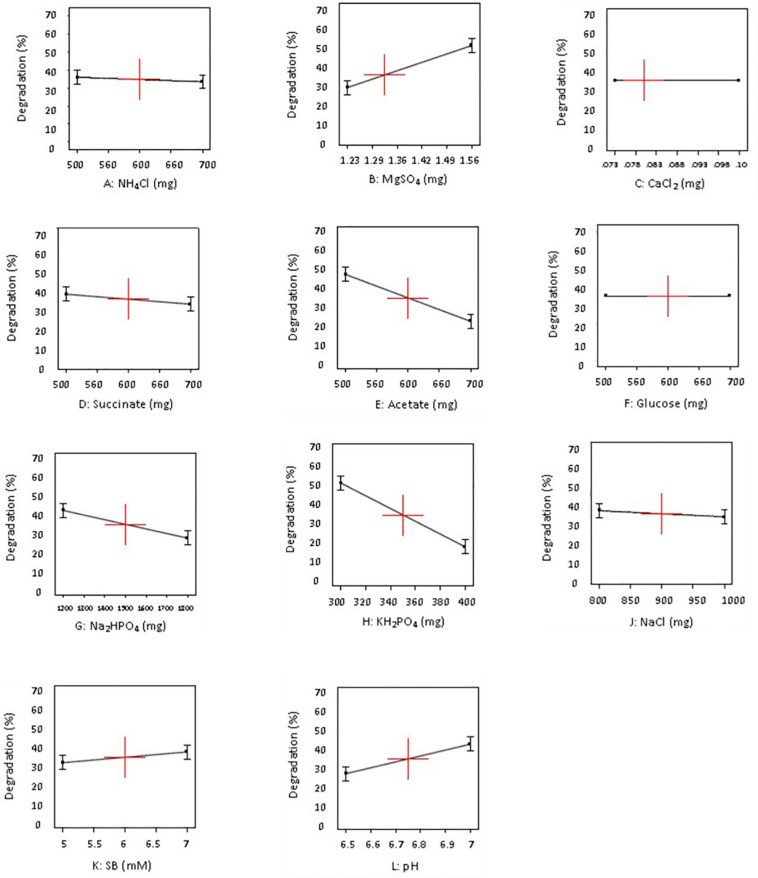
Influence of different concentrations (−1 and +1 levels) on degradation of sodium benzoate by *P. citronellolis* (design A).

**FIGURE 3:**
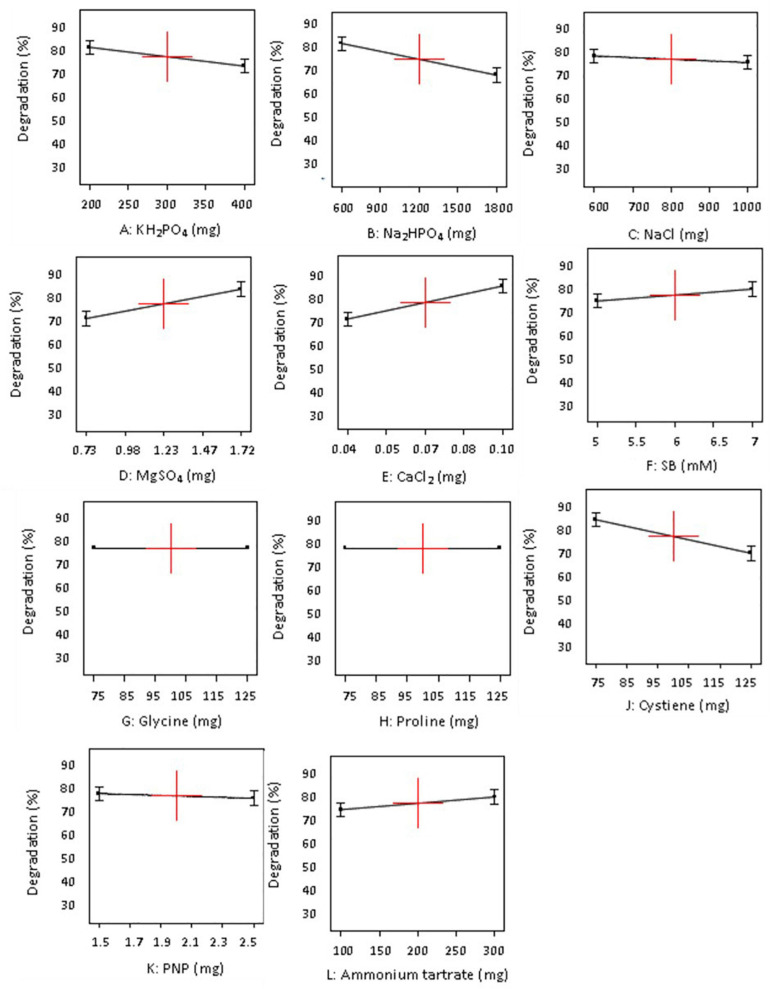
Influence of different concentrations (+ 1 and −1) of individual 11 variables on degradation of sodium benzoate by *P. citronellolis* (design B).

Table 3 depicts that levels of factors in run number 1 in design A and run number 11 in design B resulted in highest SB degradation efficiency of 69 and 86%, respectively. The significance of the experimental data generated was calculated using the Design Expert software considering the degradation percentage obtained from various wet lab experimental sets as an input parameter. Based on these experimental degradation values, the software calculates the effectiveness of different combinations of nutrients and their concentrations and generates the graphical presentation of positive and negative effects (Figures 1–3). The positive influence of factor here is described in terms of higher SB degradation achieved with increasing concentration of variable factor and *vice-a-versa* for negatively influencing factors. The Pareto charts highlighted the statistically significant factors having either positive or negative impact on SB degradation in decreasing order of influence and indicate the main effect factors by displaying them above “t critical” and Bonferroni limits (Design expert Ver. 10.0) (Figures 1A,B).

The variables with negative and positive effects on SB degradation are displayed in different colors. Out of all the parameters analyzed for study, MgSO_4_, pH, and substrate concentrations (SB) were shown to have a positive impact, of which the first two were significant for SB degradation in design A whereas MgSO_4_, CaCl_2_, ammonium tartrate, and substrate concentration (SB) in design B were shown to have a positive impact on SB degradation, among which the first two were found to be significant. On the other hand, KH_2_PO_4_, Na_2_HPO_4_, and NaCl exhibited negative effect on SB degradation in both the designs. It is important to realize that change in the selection of macronutrients did not affect the inorganic nutrients’ requirement.

The data displayed in Pareto chart became more defined in graphs presented in Figures 2, 3 when individual factors were analyzed. As seen in Figures 2, 3, the *x*- and *y*-axis in each graph represent the concentration of the factor in milligrams and percent SB degradation, respectively. The line in the graph represents the effect of each factor on SB degradation at its variable concentration (+ 1 or −1), and the slope of the line indicates relationship of degradation percentage with concentration of the variables selected for these experiments.

Likewise, in the case of design B (Figure 3), compounds like KH_2_PO_4_, Na_2_HPO_4_, NaCl, and cysteine had negative effect on degradation. On the other hand, higher levels of ammonium tartrate, MgSO_4_, CaCl_2_, and substrate concentration had positive influence on SB degradation efficiency. Whereas, in the case of glycine, proline, and PNP, in both their variable concentrations, they did not show much significant effect on SB degradation.

The ANOVA analysis of the model is represented in Table 4. The *F* value of model A was calculated as 105.48 (*p* = 0.0094) which indicated significance of the developed model. Similarly, model B was also found to be significant with *p* value of 0.0192.

**TABLE 4:** Analysis of variance for Plackett-Burman designs A and B developed for sodium benzoate degradation.

Source	Sum of squares	*df*	Mean square	*F*-value	*P*-value	Significance
Model A	9,221.39	9	1,024.60	105.48	0.0094	Significant
Model B	2,164.91	9	240.55	51.38	0.0192	Significant

Considering the coefficient values, “effect equation” for degradation of sodium benzoate was possible to be derived. The model equation for sodium benzoate degradation (*Y*) could be written as:

For design A:

(2) $$\begin{array}{c} \mathrm{Degradation}\left(Y,\mathrm{designA}\right)=39.40-1.27\times A+11.68\times \\ B-2.68\times D-13.03\times E-7.70\times G-17.73\times H-1.71\times \\ J+2.99\times K+8.20\times L. \end{array}$$

For design B:

(3) $$\begin{array}{c} \mathrm{Degradation}\left(Y,\mathrm{DesignB}\right)=75.72-3.65\times A+6.31\times \\ B-1.29\times C+5.68\times D+6.42\times E+2.31\times F-6.49\times \\ J-0.91\times K+2.46\times L. \end{array}$$

The predicted values of sodium benzoate degradation were calculated using first-order model using Design Expert (ver. 10.0) and compared with values that were determined experimentally for each run (refer to Supplementary Material). Similarity least variation observed for degradation percentage between actual and predicted values demonstrated accurate model prediction.

### Optimization of Concentrations of Major Influential Factors Using Central Composite Design and Response Surface Methodology

Steepest ascent is an experimental approach which leads us toward optimal increase in the response. The objective of such optimization processes involves the use of higher or lower concentration of significant factors to reach a maximum degradation (Montgomery, 2017). Results of both PBD contributed to narrowing down the main effect factors toward inorganic micronutrients. In both designs, KH_2_PO_4_ and Na_2_HPO_4_ displayed a negative effect. Out of these two sources of phosphate, as KH_2_PO_4_ which was proved to be above Bonferroni limit was then selected for further optimization. In the case of MgSO_4_, in both the designs, it came up to be highly positively influencing factor and thus was processed for optimization *via* RSM. Hydrogen ion concentration being the dummy factor was excluded out of the next level of experimental designing. Taking in consideration the former experiments on SB degradation in our lab and reports on NH_4_Cl being the best N source for degradation (Zaveri et al., 2015), and thus even if it was not indicated as an influencing factor, it was further selected in the CCD design. Response obtained in terms of SB degradation was analyzed, and percent degradation data is presented in Table 5.

**TABLE 5:** SB Degradation percentage achieved with CCD design using three inorganic nutrient variables.

Run	SB degradation (%)	Run	SB degradation (%)
1	96	11	97
2	97	12	97
3	97	13	97
4	97	14	98
5	97	15	97
6	97	16	98
7	97	17	98
8	97	18	98
9	98	19	0.00
10	98	20	88

Sets for all runs were performed in triplicates, and degradation percentages indicated in Table 5 represents mean values obtained. With increase in incubation time, there was an apparent increase in the degradation percentage. However, run number 19 had no change in initial sodium benzoate concentration displaying no degradation. Almost complete degradation was achieved in the rest of all sets of experiments performed.

**FIGURE 4:**
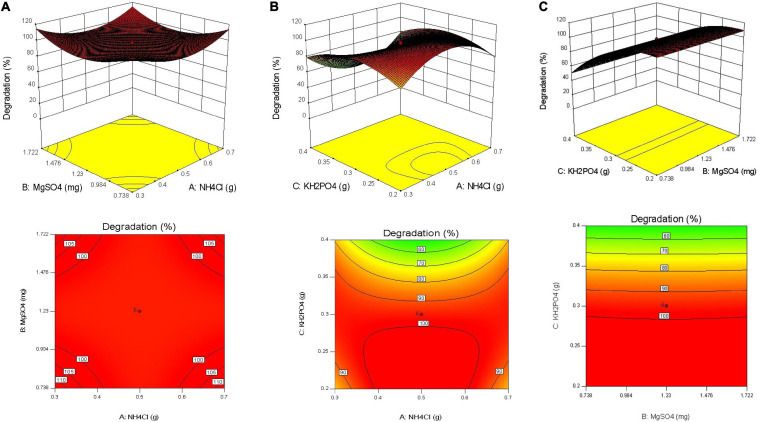
**(A)** Response surface plots and contour plots for NH_4_Cl and MgSO_4_ for sodium benzoate degradation by *Pseudomonas citronellolis*. **(B)** Response surface plots and contour plot for NH_4_Cl and KH_2_PO_4_ for sodium benzoate degradation by *Pseudomonas citronellolis*. **(C)** Response surface plots and contour plots for MgSO_4_ and KH_2_PO_4_ for sodium benzoate degradation by *Pseudomonas citronellolis*.

Response surface graphs were obtained after analysis of data. Surface plots and contour plots obtained depicted interaction of selected variables for the design (Figures 4A–C). These figures indicate responses at 24 h of incubation keeping all other parameters constant. In the case of MgSO_4_ and NH_4_Cl, all five concentrations selected were found to fall in optimally required range of concentrations for higher SB degradation. Mid-point with 100% degradation was indicated by software with relative upward boundaries of responses.

Figure 4B was found to be very informative as it clearly indicates lower concentrations of phosphate being optimal for SB degradation (≤3 mg L^–1^). Figure 4B presents interaction of NH_4_Cl and KH_2_PO_4_ at 24 h of incubation. With increase in incubation time, decline in the degradation percentage at higher concentration of KH_2_PO_4_ was observed.

Calculated values of sum of squares, *F* values, and *p* values are summarized in ANOVA table (Table 6).

**TABLE 6:** ANOVA table for central composite design for analysis of response surface obtained.

Source	Sum of squares	*df*	Mean square	*F*-value	*p*-value	Significance
Model	8,969.24	14	640.66	42.57	0.0003	Significant
A-NH_4_Cl	3.60 × 10^–4^	1	3.60 × 10^–4^	2.40 × 10^–5^	0.9963	
B-MgSO_4_	4.40 × 10^–3^	1	4.40 × 10^–3^	2.93 × 10^–3^	0.9589	
C-KH_2_PO_4_	4.75 × 10^3^	1	4.75 × 10^3^	316.17	< 0.0001	

F-value of 42.57 and p < 0.0003 proved the statistical significance of the model developed for sodium benzoate degradation by CCD design. In addition to the significance of the model, variable C was found to have a p value of less than 0.0001, indicating a significant importance of factor in design development and degradation of sodium benzoate. The calculated desirable concentration for NH_4_Cl, MgSO_4_, and KH_2_PO_4_ was found to be 0.35, 1.056, and 0.3 mg L^–1^, respectively.

## Discussion

A range of *Pseudomonas* spp. possesses a vital role in bioremediation process of textile effluents and aromatic hydrocarbon compounds. Taking the advantage of PBD design, study started with the crucial interaction of multiple carbon sources such as, acetate, succinate, and glucose and effect of micronutrients on SB degradation by *P. citronellolis*, a promising strain (Zaveri et al., 2015). The initial investigation involved the execution of 12 different run combinations for 11 variables (design A). The result of the study was remarkable and indicated a clear decrease in degradation of SB in the presence of additional carbon sources when compared with results of SB degradation where SB was present as a sole carbon source. Influence of inorganic micronutrients was also evident by results of design A performed in the presence of additional carbon sources. Results of the first PB design provoked the curiosity to understand the cumulative or individual effect of various nitrogen sources on the degradation efficiency. Another PB experiment involving 11 independent variables in 12 runs was designed (design B) using possible alternate nitrogen sources, and similar micronutrients were used as in the former design. After confirming the main effects from Pareto chart and coefficient table for significance, the concentrations of variables were further optimized by RSM.

### Effect of Alternative Carbon and Nitrogen Sources on SB Degradation—Analysis of Fractional Factorial Experiments

The result of the very first effort for understanding the interaction and influence of the presence of multiple easily assimilable carbon sources indicated an obvious negative impact on sodium benzoate degradation (Table 3). It was observed that when NH_4_Cl, MgSO_4_, CaCl_2_, Na_2_HPO_4_, and NaCl salts were at + 1 concentrations, and all the three additional carbon sources were at −1 concentrations, SB degradation achieved was maximum with a value around 69% (run number 1 in Table 3) in design A (the experimental case with carbon sources). Similar effect of biostimulation using inorganic nutrient (addition of N and P source) has been observed in case of removal of solvents from textile manufacturing wastewater (Freedman et al., 2005). Under critical environmental conditions like hypersalinity, addition of yeast extract, glucose, KCl, and four mineral nutrients (solutions of phosphate buffer, calcium chloride, magnesium sulfate, ferric chloride, ammonium sulfate) was reported to enhance phenol degradation capacity (Li et al., 2010).

It was observed that the presence of multiple carbon sources prolonged the lag phase for hydrocarbon degradation (Wigginst and Alexander, 1988). This, however, led to generation of considerable biomass but was not able to exhibit effects like co-metabolism as indicated by Schmidt and Alexander (1985). The delay as well as reduction in the efficiency of SB degradation can be attributed to the presence of additional “C” source (s) which might have been used easily before the commencement of SB degradation thus affecting the overall activity. In this experiment, the maximum SB degradation achieved was only 68.5% (Table 3), which did not support the hypothesis of faster degradation of SB as a result of higher biomass production achieved through utilization of certain easily assimilable C source.

Similarly, textile dye Remazol black B biodegradation process was optimized using the multifactorial Plackett-Burman design by Hashem et al. (2018), where 11 independent factors were included. Out of the which, eight factors were media compositions (such as glucose concentration, yeast extract, sodium acetate, sodium nitrate, EDTA, iron concentration, magnesium concentration, and NaCl) and other two were environmental conditions (temperature and pH) in addition to dye concentration. A significant increase in biodegradation rates was observed in the presence of iron, magnesium, and yeast extract and at high pH value.

Discoloration of reactive orange 4 dye was also optimized by RSM-Box-Behnken design under different cultural and nutritional conditions using *Pseudomonas putida* SKG-1 strain. Such experimental design allowed a 97.8% discoloration of dye in 72 h of incubation period. Furthermore, dye discoloration was also studied in bioreactor which gave 98% efficiency in 60 h of incubation period (Garg et al., 2015).

These observations contribute significantly to the understanding of field conditions for degradation of sodium benzoate. It is believed that degradable hydrocarbon would be taken care by the microbial community present in either effluent stream, wastewater treatment plant, soil, or sludge. The results obtained strongly stand for the organism’s behavior of selecting preferential carbon sources over hydrocarbons, and thus, leading to delayed initiation or negligible SB degradation. This indicates that the given resident time in the treatment plant or at any contaminated site may not always result in the removal of even degradable fraction of hydrocarbons or pollutants. After realization of negative influence of the presence of multiple C sources on SB degradation, another PBD experiment was designed to investigate the effects of diverse nitrogen sources (design B).

Treatment of contaminants with preferable nitrogen source could decrease lag phase and increase cell growth and bioremediation activities. Nitrogen is most often the limiting nutrient affecting biodegradation of hydrocarbon. However, it was also reported that excess amount of nitrogen source inhibits biodegradation rate in polluted soil due to osmotic soil water potential depression (Walworth et al., 2007). In the present study, it was observed that with nitrogen and minerals, lower concentration level (−1) of NaCl, Na_2_HPO_4_, cysteine, and MgSO_4_ resulted in maximum SB degradation, i.e., 87% (run number 11 in Table 3). In contrast to the observation with C sources, in the case of the second set of experiments, occurrence of multiple N sources did not influence SB degradation negatively. This study did not reveal the preferential utilization of any N source over others for SB degradation. Another observation was the absence of delay/lag in the utilization of SB as seen in the case of the first PBD design and could be achieved within 24 h optimum SB degradation.

Similar to the first PBD design, the role of inorganic micronutrients also came out to be statistically significant for SB degradation in the presence of various N sources.

### Interaction of Concentrations of Micronutrients Revealed by CCD

For next level of steep ascent focusing, two factors KH_2_PO_4_ and MgSO_4_ exhibiting main effects in Plackett-Burman design and NH_4_Cl were taken for CCD development. These experiments were aimed to meticulously optimize the addition of these nutrients for sodium benzoate degradation. Lakshmi et al. (2013) presented reports comprising optimization of phenanthrene degradation where, in addition to substrate concentration as a variable, Na_2_HPO_4_, MgSO_4_, and FeSO_4_ were found to be the most important factors for degradation. Similarly, importance and optimization of micronutrients has been emphasized in several other degradation studies using statistical tools (Mohajeri et al., 2010). Likewise, in a study focused on increasing extracellular nuclease, *Nuc*B, from *Bacillus licheniformis*, addition of manganese had a stimulating effect with a 10-fold increase in *Nuc*B activity. On the other hand, phosphate availability had an inhibitory effect on *Nuc*B synthesis (Rajarajan et al., 2013). The importance of micronutrients, magnesium and phosphate, has been emphasized for various enzyme metabolisms like enhancing the expression of amylase enzyme in *A. oryzae* CBS 819.72 (Kammoun et al., 2008) and shikimic acid production in *Citrobacter freundii* GR-21 (KC466031) (Rawat et al., 2013).

In one of the major observations, it was revealed that higher NH_4_Cl concentrations could ameliorate inhibitory effect of higher concentration of phosphate. Similar inhibitory effect of phosphate is observed in Figure 4C. Looking to the steepness of the curve at high concentration of phosphate, addition of even high concentration of MgSO_4_ may not help to increase sodium benzoate degradation. Farag et al. (2018) investigated the role of MgSO_4_ in crude oil biodegradation process by *Pseudomonas* sp. sp48 using response surface method (Box-Behnken design). This study revealed that the lower concentration of MgSO_4_ was the best for the optimal crude oil biodegradation process.

Contour of response displayed a step-wise change in colors toward green, indicating a decrease in the degradation percentage with increase in KH_2_PO_4_ concentrations. Similarly, diesel oil degradation was optimized using PB and RSM (CCD) where three parameters, i.e., P source (KH_2_PO_4_), pH, and N source (NaNO_3_) were major influencers affecting oil degradation. It was observed that middle range of KH_2_PO_4_ concentration (0.022 g) was sufficient for the ideal diesel biodegradation. The optimal diesel oil biodegradation (total petroleum hydrocarbons 125 mg L^–1^) was obtained with 0.143 g of NaNO_3_, 0.22 g of KH_2_PO_4_, and at 7.4 pH. These results demonstrated the cellular levels of C, N, and P ratios required for the significant biodegradation rate (Xia et al., 2012). This observation would help in modulating the concentration of required nutrient in field conditions while facing shock loads of phosphate. Such detailed analysis of bioprocess and optimization may lead to unfolding of more interaction of nutrient and may help in improving the performance of laboratory-optimized studies in the field.

The next question arises for the type of pathway and the mechanism involved for sodium benzoate degradation available with *P. citronellolis* which may enhance our understanding about the complete role of nutrients in the regulation of the degradation process. The molecular details of generalized pathway utilized by aerobic bacteria are widely known and are available with KEGG database. However, the associated mechanism for sodium benzoate degradation by *P. citronellolis* is being explored by studying specific enzymatic reactions, transcriptome analysis, and gene expression studies.

## Conclusion

Statistical methods, PB and RSM enabled to understand the interplay of additional carbon and nitrogen sources as well as micronutrients on sodium benzoate degradation by *P. citronellolis.* Being vulnerable to biological degradation, even sodium benzoate degradation faced severe competition due to the co-existence of other carbon sources. Micronutrients turned out to be the major influencing factors, of which NH_4_Cl and MgSO_4_ were positively influencing whereas KH_2_PO_4_ was negatively influencing sodium benzoate degradation. However, the negative effects of KH_2_PO_4_ could be nullified by higher amount of NH_4_Cl. Statistically significant model was developed for SB degradation where the desirable concentrations of micronutrients were derived. With the ability to quantitatively describe the interaction effects of multiple nutrients on the system response, DOE and statistical modeling helps in strategically designing bioprocesses for successful full-scale application of bioremediation.

## Data Availability Statement

The datasets presented in this study can be found in online repositories. The names of the repository/repositories and accession number(s) can be found in the article/Supplementary Material.

## Author Contributions

NM and PZ conceptualized the research work. PZ executed in laboratory, prepared the draft of manuscript. NM edited the manuscript. RP and AI helped in performing the statistical and data analysis as well as in preparation of manuscript. All authors contributed to the article and approved the submitted version.

## Conflict of Interest

The authors declare that the research was conducted in the absence of any commercial or financial relationships that could be construed as a potential conflict of interest.
