# Supplementary material for: Uncovering Competitive and Restorative Effects of Macro- and Micronutrients on Sodium Benzoate Biodegradation
Source: Front Microbiol. 2021 Mar 17;12:634753. doi: 10.3389/fmicb.2021.634753 (PMC8009979; doi:10.3389/fmicb.2021.634753)
Supplement: Supplementary file 1 [file Data_Sheet_1.docx]

**Supplementary Material to:**

Zaveri P, Iyer A, Patel R and Munshi N “Uncovering competitive and restorative effects of macro - and micro – nutrients on sodium benzoate biodegradation”.

3 Pages, 3 Tables

**Table S1 (a)**: Combination of variables with “C” sources and micronutrients in Plackett Burman design ‘A’.

| **Run** | **A: NH_4_Cl** | **B: MgSO_4_** | **C: CaCl2** | **D: Succinate** | **E: Acetate** | **F: Glucose** | **G: Na_2_HPO_4_** | **H: KH_2_PO_4_** | **J: NaCl** | **K: SB** | **L: pH** |
| --- | --- | --- | --- | --- | --- | --- | --- | --- | --- | --- | --- |
|  |  |  |  |  |  |  |  |  |  |  |  |
| 1 | +1 | +1 | +1 | -1 | -1 | -1 | +1 | -1 | +1 | +1 | -1 |
| 2 | -1 | +1 | +1 | +1 | -1 | -1 | -1 | +1 | -1 | +1 | +1 |
| 3 | +1 | +1 | -1 | +1 | +1 | +1 | -1 | -1 | -1 | +1 | -1 |
| 4 | +1 | +1 | -1 | -1 | -1 | +1 | -1 | +1 | +1 | -1 | +1 |
| 5 | -1 | +1 | +1 | -1 | +1 | +1 | +1 | -1 | -1 | -1 | +1 |
| 6 | -1 | -1 | -1 | +1 | -1 | +1 | +1 | -1 | +1 | +1 | +1 |
| 7 | +1 | -1 | +1 | +1 | -1 | +1 | +1 | +1 | -1 | -1 | -1 |
| 8 | +1 | -1 | +1 | +1 | +1 | -1 | -1 | -1 | +1 | -1 | +1 |
| 9 | -1 | -1 | -1 | -1 | -1 | -1 | -1 | -1 | -1 | -1 | -1 |
| 10 | -1 | +1 | -1 | +1 | +1 | -1 | +1 | +1 | +1 | -1 | -1 |
| 11 | +1 | -1 | -1 | -1 | +1 | -1 | +1 | +1 | -1 | +1 | +1 |
| 12 | -1 | -1 | +1 | -1 | +1 | +1 | -1 | +1 | +1 | +1 | -1 |

**Table S1 (b)**: Combination of variables with “N” sources and micronutrients in Plackett Burman design ‘B’.

| **Run** | **A: KH_2_PO_4_** | **B: Na_2_HPO_4_** | **C: NaCl** | **D: MgSO_4_** | **E: CaCl_2_** | **F: Sodium Benzoate** | **G: Glycine** | **H: Proline** | **J: Cystiene** | **K: PNP** | **L: Ammonium tartrate** |
| --- | --- | --- | --- | --- | --- | --- | --- | --- | --- | --- | --- |
|  |  |  |  |  |  |  |  |  |  |  |  |
| 1 | -1 | +1 | +1 | -1 | +1 | +1 | +1 | -1 | -1 | -1 | +1 |
| 2 | -1 | -1 | +1 | -1 | +1 | +1 | -1 | +1 | +1 | +1 | -1 |
| 3 | -1 | +1 | +1 | +1 | -1 | -1 | -1 | +1 | -1 | +1 | +1 |
| 4 | -1 | -1 | -1 | -1 | -1 | -1 | -1 | -1 | -1 | -1 | -1 |
| 5 | +1 | +1 | -1 | +1 | +1 | +1 | -1 | -1 | -1 | +1 | -1 |
| 6 | +1 | +1 | -1 | +1 | +1 | -1 | +1 | +1 | +1 | -1 | -1 |
| 7 | +1 | -1 | +1 | +1 | +1 | -1 | -1 | -1 | +1 | -1 | +1 |
| 8 | +1 | +1 | +1 | -1 | -1 | -1 | +1 | -1 | +1 | +1 | -1 |
| 9 | +1 | +1 | -1 | -1 | -1 | +1 | -1 | +1 | +1 | -1 | +1 |
| 10 | +1 | -1 | +1 | +1 | -1 | +1 | +1 | +1 | -1 | -1 | -1 |
| 11 | +1 | -1 | -1 | -1 | +1 | -1 | +1 | +1 | -1 | +1 | +1 |
| 12 | -1 | -1 | -1 | +1 | -1 | +1 | +1 | -1 | +1 | +1 | +1 |

**Table S2:** Details of concentrations used for Central Composite Design for response surface analysis.

| **Run** | **Factor 1** | **Factor 2** | **Factor 3** |
| --- | --- | --- | --- |
|  | **A: NH_4_Cl** | **B: MgSO_4_** | **C: KH_2_PO_4_** |
| 1 | 0 | 0 | 0 |
| 2 | +1 | -1 | +1 |
| 3 | +1 | -1 | -1 |
| 4 | 0 | 0 | 0 |
| 5 | 0 | 0 | 0 |
| 6 | -1.68 | 0 | 0 |
| 7 | +1.68 | 0 | 0 |
| 8 | 0 | 0 | 0 |
| 9 | 0 | 0 | 0 |
| 10 | 0 | -1.68 | 0 |
| 11 | +1 | +1 | +1 |
| 12 | +1 | +1 | -1 |
| 13 | -1 | -1 | +1 |
| 14 | -1 | -1 | -1 |
| 15 | 0 | 0 | -1.68 |
| 16 | -1 | +1 | -1 |
| 17 | 0 | +1.68 | 0 |
| 18 | -1 | +1 | +1 |
| 19 | 0 | 0 | +1.68 |
| 20 | 0 | 0 | 0 |
